# A New Design of Tubular Ceramic Membrane Module for Oily Water Treatment: Multiphase Flow Behavior and Performance Evaluation

**DOI:** 10.3390/membranes10120403

**Published:** 2020-12-07

**Authors:** Guilherme L. Oliveira Neto, Nívea G. N. Oliveira, João M. P. Q. Delgado, Lucas P. C. Nascimento, Ricardo S. Gomez, Adriano S. Cabral, Daniel C. M. Cavalcante, Vansostenes A. M. Miranda, Severino R. Farias Neto, Antonio G. B. Lima

**Affiliations:** 1 Federal Institute of Education, Science and Technology of Piauí, Floriano 64808-475, Piauí, Brazil; guilherme@ifpi.edu.br; 2 Technical School of Floriano, Federal University of Piauí, Floriano 64808-605, Piauí, Brazil; niveagomes@ufpi.edu.br; 3 CONSTRUCT-LFC, Department of Civil Engineering, Faculty of Engineering, University of Porto, 4200-465 Porto, Portugal; jdelgado@fe.up.pt; 4 Department of Mechanical Engineering, Federal University of Campina Grande, Campina Grande 58429-900, Paraíba, Brazil; lucaspereira.cn@hotmail.com (L.P.C.N.); swr140@gmail.com (A.S.C.); antonio.gilson@ufcg.edu.br (A.G.B.L.); 5 Federal Institute of Education, Science and Technology of the Sertão Pernambucano, Serra Talhada 56915-899, Pernambuco, Brazil; danielcesar_fisico@yahoo.com.br; 6 Federal Institute of Education, Science and Technology of Pernambuco, Belo Jardim 55145-065, Pernambuco, Brazil; vansostenes.miranda@belojardim.ifpe.edu.br; 7 Department of Chemical Engineering, Federal University of Campina Grande, Campina Grande 58429-900, Paraíba, Brazil; severino.rodrigues@professor.ufcg.edu.br

**Keywords:** produced water, membrane filtration, computational fluid dynamics, ANSYS Fluent

## Abstract

Petroleum has been extracted from oil reservoirs using different techniques. This activity is accompanied for a large amount of water and sometimes mixed with gas. This produced water has a high oil concentration and other toxic chemical compounds, thus, it must be treated to be reused or released to environment according to environmental protection regulations. Currently, ceramic membrane technology has been employed in the wastewater treatment, due to its high benefit–cost ratio. In this sense, this work aims to study the oil–water mixture separation process using a new configuration of tubular ceramic membrane module by computational fluid dynamic (ANSYS Fluent software). The proposed model is composed of mass and linear momentum conservation equations coupled to Darcy’s law and SST k-ω turbulence model. Results of the volumetric fraction, pressure, and velocity distribution of the oil and water phases are presented and discussed. The results indicated that the proposed model and new device both have great potential to be used on the water/oil separation process and that the transmembrane pressure remains constant in the axial direction and decreases radially through the membranes, indicating an efficient system that favors the transport of clean water and oil retention.

## 1. Introduction

Produced water is the effluent from the oil and gas industry emitted in large quantities when water from underground reservoirs is brought to the surface during activities at sea or on land. It is a large amount of residual water with different characteristics, which carries different contaminants, mainly due to the variation of organic and inorganic compounds from the wells [1,2], featuring an extremely effluent saline, oily and toxic to living beings. It is estimated that for every 1 barrel of oil produced, about 3 barrels of residual water are released [3]. According to Igunnu and Chen [4], approximately 250 million barrels of water produced are generated daily from the oil and gas fields, and more than 40% of this effluent is released into the environment.

However, the spill of produced water or its discharge on the surface, without previous treatments or treated improperly, can contaminate water resources and generate health concerns, mainly due to the high load of chemical compounds (including heavy metals), extremely saline, slight corrosive potential, in addition to compounds that form from spontaneous reactions, such as brominated compounds, derived from bromide [1,2,4].

Currently, there are strict regulations that oblige oil and gas companies to apply different methods for the treatment of produced water, aiming at its decontamination in such a way that the treated water (or final permeate) is discarded or reused without environmental risks or for health [1,2,5]. In Brazil, the National Environment Council (CONAMA) is the main regulator of effluent release conditions and standards, establishing a maximum oil and grease content of 20 mg/L for disposal [6]. In this context, [1] reported that the ideal method for the treatment of produced water must be selected according to the main constituents of the effluent, as well as environmental factors, economic factors, and local regulatory structure. The technologies applied to treat the resulting water are generally based on physical, physical-chemical, chemical, and biological principles, applied through the processes of flocculation, coagulation, filtration, biological treatments, hydrocycloning, and different types of separation membranes [1,7]. Notably, many of these wastewater treatment processes are complex, expensive, slow, difficult to maintain and high energy consumption [8].

According to Jepsen et al. [9], traditional technologies for the treatment of produced water (such as the use of hydrocyclones) are already working within their fundamental limit, so new filtration technologies must be proposed in order to enhance water purification. In this context, membrane technologies, with a great emphasis on ceramic membranes, present excellent chemical, thermal, and mechanical properties, favoring high performance in severe operating conditions, such as high temperatures and the presence of aggressive chemicals [10]. Another advantage of membrane technology is related to the large volume of treated water: Even with a high load of dispersed solids and oily contaminants without necessarily adding chemicals, the membranes can be washed, offering reliable performance for long periods of time. Thus, membrane separation has become more efficient compared to conventional techniques, and this efficiency depends entirely on the properties of the membrane [11].

Membranes can be classified as polymeric or ceramic [12]. Both can be manufactured in a wide variety of configurations, leading to different degrees of separation [11].

Ceramic membranes have a useful life of more than 10 years and do not require chemical products during the treatment of produced water, except during periodic cleaning of the membranes themselves [4]. The use of ceramic membranes to treat oily effluents has grown considerably, from organic membranes affected by polar solvents, chlorinated solvents and high oil fraction. The results are extremely satisfactory, but problems such as fouling formation and concentration polarization are recurrent, decreasing the permeate flow and compromising the efficiency of the membranes.

Sieving, adsorption and bridges are the main mechanisms by which ceramic membrane filtration occurs. The screening takes place for particles, micro-organisms and organic molecules whose size is larger than the size of the membrane pores; these are retained on the surface. The adsorption follows the principles of van der Waals force, chemical bonding force or electrostatic attraction, through which the contaminants are retained on the membrane surface and pores, even the small particles. In turn, bridges occur due to the interaction between pollutants, which unite in an entire body and are intercepted by the ceramic membrane [13].

In the oily water separation process, the forces exerted by pressure, adsorption, and electric charge cause deformation of oil particles with small diameter in such a way that these particles cannot pass through the larger pores in the membrane. This phenomenon occurs because the organic phase (oily phase) in the liquid–liquid dispersion system is separated from the aqueous phase by means of lipophilic or hydrophilic properties of the porous membrane. Knowing that the two liquids are not miscible with each other and that there are differences in the affinity of the membrane for the different liquids, one of these liquids will form a pure liquid layer of a certain thickness on the membrane surface under hydraulic and external forces. Thus, the concentration of the other liquid forms a gradient with the pure liquid layer that stimulates their separation [13].

The concentration polarization phenomenon is established from the formation of an oil layer and other contaminants from the effluent parallel to the membrane surface [14,15]. The increase in the concentration of these retained materials favors a concentration gradient that causes additional resistance to mass transfer across the membrane [16]. In fact, this polarization by concentration of solute on the membrane surface is the primary reason for the decline in flow during the initial period of a membrane separation process. This can occur in conjunction with irreversible incrustations in the membrane and/or reversible formation of an encrustation layer [17].

Increasing the speed of the effluent flow is one of the strategies to enhance the mass transfer rate and decrease polarization by concentration. This effect was clearly observed by Cui et al. [18], when they applied ceramic microfiltration membranes combined with zeolite to treat water contaminated with oil. The authors observed that a higher tangential flow velocity (0.01 m/s) doubled the flow through the membrane without affecting the oil rejection coefficient. In addition, the membrane performance remained excellent, even with high oil concentration in the feed solution (500 mg/L).

Membrane encrustation generally occurs as a result of adsorption of components from the feed solution to the membrane surface [19]. This encrustation reduces the membrane separation performance, but the occurrence of this phenomenon can be mitigated by modifying the membrane surface. For example, Zhou et al. [20] modified commercial microfiltration membranes based on Al_2_O_3_ by coating with nanometric-sized ZrO_2_, to reduce clogging of the membrane by oil droplets. The constant flow of the modified membrane was obtained in a very short time, maintaining 88% of the initial flow rate and oil rejection above 97.8%. In addition, the combination of this coating, with the appropriate backwash and transverse flow rate, helps to reduce the obstruction of the membrane by oil droplets.

Another effective method to delay polarization by concentration, reduce fouling and, consequently, increase the permeation flow is the use of pulsed flow and the promotion of turbulent flow [15,21]. Despite the problems with polarization by concentration and fouling, ceramic membranes are more resistant to contamination than organic ones, when it comes to petrochemical effluents [15]. All this potential of ceramic membranes justifies the intensification of scientific works that seek to enhance the treatment of water produced using this device.

Ceramic membranes have high filtration efficiency due to their excellent hydrophilic properties and chemical and hydrothermal stability [13]. Ceramic membranes are considered an economical and promising technology for water treatment and purification due to their ability to achieve almost 100% rejection of dissolved solids. This is due to the fact that the membranes are composed of components with hydrophobic properties (such as Silane agents), which favor only the transport of vapor, while retaining the unwanted solutes in the liquid phase on the feed side [22].

The resistance of the membrane itself is one of the factors that influence the flow performance through the membrane and, necessarily, must be considered, as it will always exist from the beginning to the end of the filtration process. This resistance is due to geometric factors, such as pore size and distribution, thickness and affinity of the membrane surface with the solvent [14,19].

Based on the above, the separation process by ceramic membranes can be understood as being dynamic for different characteristics/constitution of oily effluents and the membrane itself. In addition, the wide variation in several operational parameters results in a different filtration potential and must also suit the specific properties of each effluent and type of membrane in operation. Because the great importance, membrane separation processes have been studied on the experimental and theoretical approaches. Under the theoretical point of view, the application of computational fluid dynamics (CFD) techniques has advanced technologically and helped in the understanding and development of new membrane separation processes, but problems such as fouling, concentration polarization, and pore clogging, which reduce the filtration rate and favor the contamination of the permeate, remain challenges to be overcome [23,24,25,26,27,28,29].

In this context, the construction of new design of ceramic membranes, especially with adaptations of the mesh and pore arrangement emerges as one of the first steps for the development of new prototypes of practical and efficient application in the treatment of produced water. In addition to the development of new membrane models, the simulation of the filtration process is essential for establishing the operational characteristics, understanding and maximizing the filtration process. In this premise, it is worth highlighting the behavior of the pressure distribution, velocity and concentration field of the components in the separation module as well as the porosity and permeability in the membrane.

Therefore, in addition to the works reported in the literature, this research aims to study the separation process of produced water (oily water) using a tubular ceramic membrane module and the computational fluid dynamics (CFD) technique. The idea is to evaluation the separation performance and fluid flow behavior inside module and membranes. The following innovative aspect of this research can be highlighted: The study focuses in a new design of the membrane module and in the robustness of the proposed mathematical model that includes coupled phenomena of turbulent multiphase flow inside the module shell and membrane (wall thickness and tubular region), not yet evaluated in the literature.

## 2. Methodology

### 2.1. Description of the Domain under Study

**Figure 1:**
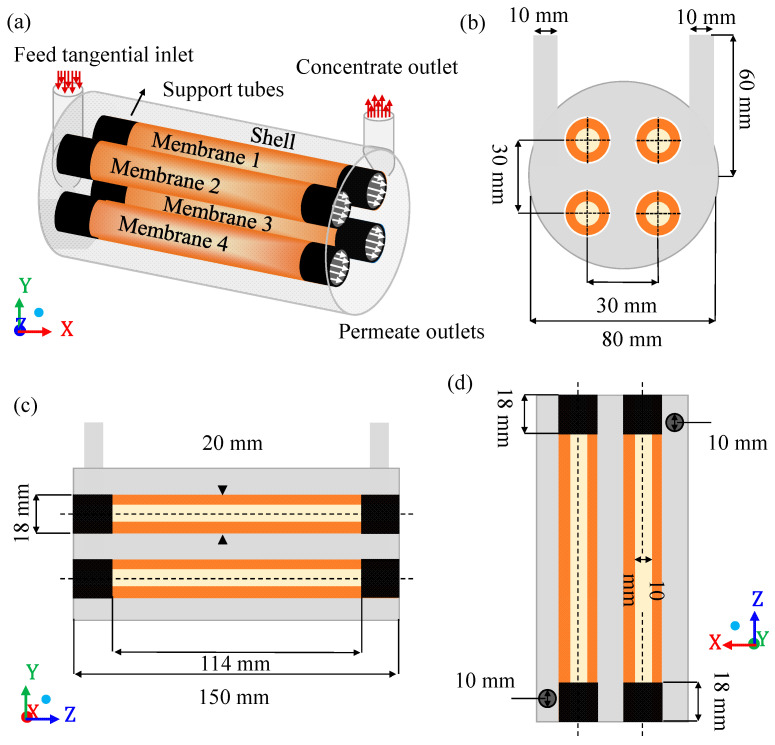
Geometric representation of the module under study (**a**); front view of the separator (**b**); side view of the separator (**c**), and top view of the separator (**d**).

In this study, a shell-tube ceramic membrane filtration module (domain under study) was proposed, which consists of a main cylinder (hull) equipped with a tangential inlet and outlet and four internal cylinders corresponding to the membranes porous ceramics (membranes 1, 2, 3, and 4), as shown in Figure 1.

The separation module acts through nine structural domains, namely: The four volumes of fluids related to the four membranes, the four permeate (internal part of the membrane) and the cylindrical hull, comprised between the internal surfaces, which limit the separator and the outer surfaces of the membranes. The geometric dimensions of this module, used in numerical simulations, are described in Table 1.

The module entrance is a tangential circular tube through which the contaminated effluent enters the system. The outlet tube, also tangential circular, is intended for the outlet of the concentrate. The filtrate is retained inside the membranes after filtration. The fact that the fluid flow in the membrane is considered not yet included in the literature for this type of separator makes the proposal of this research innovative. Thus, the separation between oil and water occurs as the feed mixture, containing a certain oil concentration, enters tangentially into the separator. Mainly due to the difference in transmembrane pressure, the mixture is forced to cross the membranes and, depending on the operational conditions and membrane characteristics, the oil fraction is retained in the porous structure, and only the aqueous phase easily crosses the membrane, generating the permeate. Finally, the four outlets coming from the membranes receive the permeate flow, while the outlet in the hull receives the concentrate (mixture of water with high oil concentration).

### 2.2. Computational Mesh

For a numerical solution of the conservation equations that govern the domain under study, it is necessary to convert the continuous domain into a discrete domain. These control volumes are three-dimensional partitions of the geometric domain, composed of edges and nodal points, in which the discretized governing equations must be solved, to predict the physical phenomenon. The numerical method causes errors of truncation and idealizations, which decrease, as the mesh build with high number of elements, that is, as the finite limits of solution are reduced in each control volume.

To adequately predict the proposed problem, three hybrid meshes (tetrahedral and hexahedral elements) with different densities of control volumes (elements) were developed, using the ANSYS Meshing^®^ 15.0 software (Canonsburg, PA, USA), as shown in Table 2. For the hull domain, hexahedral elements close to the outer walls of the membranes and the hull itself were built, and tetrahedral elements were built for the other regions of the study domain. In the membranes, structured meshes were built with only hexahedral elements, which was maintained for the cylindrical domains, referring to the permeate volumes. This procedure was realized using the mesh construction technique named *o-grid.*

Table 2 shows the parameters of mesh quality, orthogonality (minimum and average), deformation (maximum and average) and number of elements, referring to the three meshes, with 218,704, 508,325, and 853,536 elements, developed throughout the research. It is observed, from the ANSYS Meshing^®^ software, that all meshes are within the recommended limits for values of deformation (below 0.95) and orthogonality (above 0.1). These meshes were evaluated for the mesh dependence study in relation to the number of elements.

**Figure 2:**
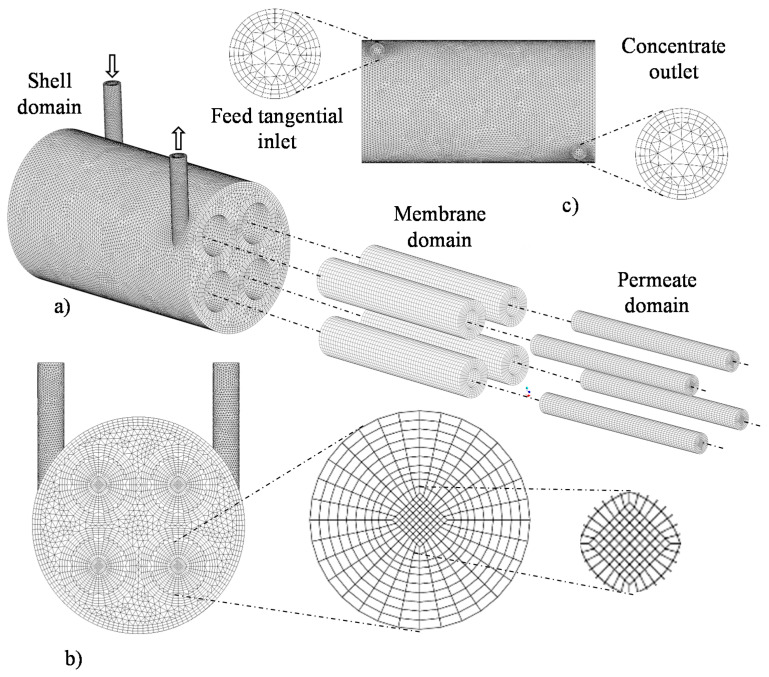
Mesh 2 of the separation module, highlighting the hull, membranes and permeate regions (**a**), front view (**b**) and top view (**c**), used during the computational fluid dynamics (CFD) simulation.

**Figure 3:**
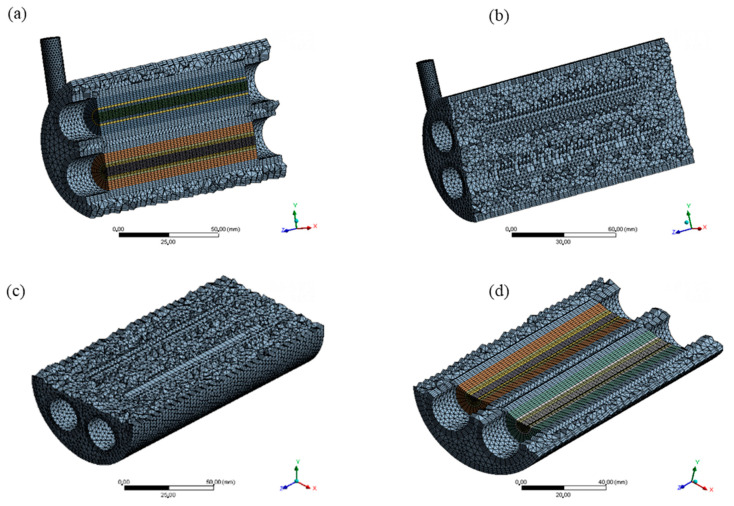
Views of the mesh in vertical longitudinal sections (**a**,**b**) and transversal longitudinal sections (**c**,**d**).

Figure 2 and Figure 3 illustrate aspects of mesh 2, with emphasis on the studied domains. Especially in Figure 2, it is possible to observe details in the mesh of the hull, membranes and permeate domains. There is also a cross-section of the entire module, focusing on the membrane mesh, as well as the mesh entrance and exit regions.

Figure 3 shows an internal view of the mesh, detailed from vertical longitudinal and transversal longitudinal sections. In this way, it is possible to visualize clearly the internal mesh of the hull, membranes and permeate domains.

### 2.3. Mathematical Modeling

#### 2.3.1. Multiphase Model for the Fluid Medium

The Eulerian–Eulerian multiphase model is capable of modeling multiple phases, treated as separate, yet interactive. The phases can be liquid, gaseous, or solid, in almost any combination. A Eulerian–Eulerian solution treatment is used for each phase separately, even if one of the phases is made up of particles. The model makes no distinction between fluid–fluid and fluid–solid (granular) flow. A single pressure is shared by all phases, and the linear momentum and continuity equations are solved for each phase [30].

The formulation of the model assumes that two or more phases are continuous and interpenetrable. For each phase added to the mathematical model, a variable is introduced: The volumetric fraction ($\alpha_{q}$), of the respective phase “q”. The volumetric fractions represent the volume occupied by each phase, and the mass and linear momentum conservation laws are satisfied for each phase individually. In each control volume (V), the sum of the volumetric fractions of the phases is equal to 1 (one), as follows.

(1) $$V_{q}=\overset{}{\underset{V}{\int }}\alpha_{q}\mathrm{dV},$$

where:

(2) $$\overset{n}{\underset{q=1}{\sum }}\alpha_{q}=1.$$

The effective density of phase q is defined by:

(3) $${\hat{\rho }}_{q}=\alpha_{q}\rho_{q},$$

where $\rho_{q}$ is the physical density of phase q.

Thus, there are three possibilities:

$\alpha_{q}=1$: Indicates that the volume is completely filled by the q phase.

$\alpha_{q}=0$: Indicates that the volume does not even have a region filled by the q phase.

$0<\alpha_{q}<1$: Indicates that the volume has an interface between the q phase and one or more phases.

Based on the local value of $\alpha_{q}$, of the n phases existing in the physical process, the properties and the appropriate variables are weighted in each region of the multiphase flow. In this work, it was considered that the mass and linear momentum conservation equations are solved for each of the present phases (continuous and dispersed). For each of the situations, the following considerations were adopted:

- Newtonian and incompressible fluid;
- Flow in permanent and isothermal regime;
- Mass transfer, interfacial momentum and mass sources were neglected;
- The non-drag interfacial forces (lift forces, wall lubrication, virtual mass, turbulent dispersion, and solid pressure) were neglected;
- There is no transfer of interfacial mass;
- The module walls are static and null roughness;
- The water stream is considered to be a mixture of water and oil;
- The viscosity and density of the mixture and other physical and chemical properties are constant;
- The porous medium (ceramic membrane) has an isotropic distribution of pores and permeability; and
- There is no reaction or adsorption of the solute on the contact surface in the porous medium.

From the above assumptions, the mass conservation equation for multiphase flow was defined by Equation (4), as follows:

(4) $$\nabla \cdot \left(\alpha_{q}\rho_{q}{\vec{v}}_{q}\right)=0,$$

where the sub-index “q” represents the phase involved in the two-phase water/oil mixture; α, ρ and $\vec{v}$ are the volumetric fraction, density, and velocity vector, respectively.

In turn, the linear momentum conservation equation for multiphase flow was defined by Equation (5), given below:

(5) $$\nabla \cdot \left(\alpha_{q}\rho_{q}{\vec{v}}_{q}{\vec{v}}_{q}\right)=-\alpha_{q}\nabla P+\nabla \cdot {\overset{=}{\tau }}_{q}+\alpha_{q}\rho_{q}\vec{g}+\overset{n}{\underset{p=1}{\sum }}{\vec{R}}_{\mathrm{pq}},$$

where ${\overset{=}{\tau }}_{q}$ is the stress-tensor for the q phase and ${\vec{R}}_{\mathrm{pq}}$ is the term for the interface forces. This force depends on friction, pressure, cohesion and other effects and is subject to the following condition:

(6) $${\vec{R}}_{\mathrm{pq}}={\vec{\;R}}_{\mathrm{qp}}\;\mathrm{and}\;{\vec{R}}_{\mathrm{qq}}=0.$$

The Fluent software solver uses a simplified interaction term, as follows:

(7) $$\overset{n}{\underset{p=1}{\sum }}{\vec{R}}_{\mathrm{pq}}=\overset{n}{\underset{p=1}{\sum }}K_{\mathrm{pq}}\left({\vec{v}}_{p}-{\vec{v}}_{q}\right),$$

where $K_{\mathrm{pq}}=K_{\mathrm{qp}}$ is the interfacial coefficient of momentum transfer; ${\vec{v}}_{p}$ and ${\vec{v}}_{q}$ are the velocities of the p and q phases.

For a two-phase flow, it is assumed that the secondary phase is in the form of drops. This has an impact on how each fluid is assigned to each phase, for example: In flows where there are unequal quantities of two fluids, the predominant fluid must be modeled as the primary fluid, since the sparse fluid is more likely to form droplets or bubbles [31]. The exchange coefficient for bubbling, liquid–liquid or gas-liquid mixtures can be written as follows:

(8) $$K_{\mathrm{pq}}=\frac{\rho_{p}f}{6\tau_{p}}d_{p}A_{i},$$

where $A_{i}=\frac{6\alpha_{p}\left(1-\alpha_{p}\right)}{d_{p}}$ is the interfacial area, f is the drag function, which is defined according to the exchange rate model used, and the term $\tau_{p}$ is the “particulate relaxation time”, defined as follows:

(9) $$\tau_{p}=\frac{\rho_{p}d_{p}{}^{2}}{18\mu_{q}},$$

where $d_{p}$ is the diameter of the drop.

To determine the drag function f, the Schiller and Naumann model, was used, as follows:

(10) f=CDRe24,

where $C_{D}$, is the drag coefficient, given by:

(11) CD={24(1+0.15Re0.687)/Re,Re≤1000  0.44,Re>1000,

with Re being the relative Reynolds number, defined for the primary phase q and for the secondary phase p, as follows:

(12) Re=ρp|v→p−v→q|dpμq.

To describe the turbulent flow within the system, the SST k-ω turbulence model was used. To activate the equations of the standard k-ω and k-ε turbulence models, coupling functions are used in cells near and far from the walls, respectively. The variable k represents the turbulent kinetic energy of flow, while the variable ω represents the energy dissipation rate. The transport of these variables is described by Equations (13) and (14).

(13) $$\frac{\partial \left(\rho k\right)}{\partial t}+\frac{\partial \left({\rho \mathrm{ku}}_{i}\right)}{\partial X_{i}}=\frac{\partial }{\partial X}\left(\Gamma_{k}\frac{\partial k}{\partial X_{j}}\right)+G_{k}-Y_{k}+W_{k\;},$$

and

(14) $$\frac{\partial \left(\rho \omega \right)}{\partial t}+\frac{\partial \left({\rho \omega u}_{i}\right)}{\partial X_{i}}=\frac{\partial }{\partial X}\left(\Gamma_{\omega }\frac{\partial \omega }{\partial X_{j}}\right)+G_{\omega }-Y_{\omega }+D_{\omega }+W_{\omega },$$

where t is time, X is the position vector, $u_{i}$ is the velocity vector, sub-indices i and j represent the components (x, y, and z) of the coordinate axes, so that, if i or j = 1, one has the component in the x-direction; if i or j = 2, we have the component in the y-direction, if i or j = 3, we have the component in the z-direction.

In addition, the term $G_{k}$ represents the production of turbulent kinetic energy, $G_{\omega }$ represents the generation of ω, and $\Gamma_{k}$ and $\Gamma_{\omega }$ are the effective diffusivity of k and ω, respectively, due to turbulence. The variables $Y_{k}$ and $Y_{\omega }$, represent the dissipation of k and ω, the term $D_{\omega }$ represents the cross diffusion, and the terms $W_{k\;}$ and $W_{\omega }$ are the source terms of the referred equations.

The effective diffusivities of k and ω ($\Gamma_{k}$ and $\Gamma_{\omega }$) are given by Equations (15) and (16), as follows:

(15) $$\Gamma_{k}=\mu +\frac{\mu_{t}}{\sigma_{k}},$$

and

(16) $$\Gamma_{\omega }=\mu +\frac{\mu_{t}}{\sigma_{\omega }},$$

where $\sigma_{k}$ and $\sigma_{\omega }$ are the turbulent Prandtl numbers given by Equations (17) and (18), below:

(17) $$\sigma_{k}=\frac{1}{\frac{F_{1}}{\sigma_{k,1}}+\frac{\left(1-F_{1}\right)}{\sigma_{k,2}}},$$

and

(18) $$\sigma_{\omega }=\frac{1}{\frac{F_{1}}{\sigma_{\omega ,1}}+\frac{\left(1-F_{1}\right)}{\sigma_{\omega ,2}}}.$$

In the Equations (17) and (18), $F_{1}$ is one of the coupling functions between the models k-ω and k-ε, described in Equation (19), and $\sigma_{k,1}$, $\sigma_{k,2}$, $\sigma_{\omega ,1}$, and $\sigma_{\omega ,2}$ are constants of the model which are described in Table 3.

The $F_{1}$ coupling function is given by:

(19) $$F_{1}=\tan h\left(\theta_{1}{}^{4}\right),$$

where $\theta_{1}$ is determined by Equation (20).

(20) $$\theta_{1}=\min\left[\max\left(\frac{\sqrt{k}}{0.09\omega d_{g}},\frac{500\mu }{\rho d_{g}{}^{2}\omega }\;\right),\frac{4\rho k}{\sigma_{\omega ,2}D_{\omega }^{+}d_{g}{}^{2}}\right],$$

being $d_{g}$ the distance between the position studied in the domain and the nearest wall. In turn, $D_{\omega }^{+}$, is calculated by Equation (21).

(21) $$D_{\omega }^{+}=\max\left[2\rho \frac{1}{\sigma_{\omega ,2}}\frac{1}{\omega }\frac{\partial k}{\partial X_{j}}\frac{\partial \omega }{\partial X_{j}},10^{-10}\right].$$

The term $\mu_{t}$ in the Equations (15) and (16) is the turbulent viscosity given as follows:

(22) $$\mu_{t}=\frac{\rho k}{\omega \cdot \max\left[\frac{1}{\alpha^{*}},\frac{{\mathrm{SF}}_{2}}{\alpha_{1}\omega }\;\right]},$$

where $\alpha_{1}$ = 0.31 [30] and $\alpha^{*}$ is the damping factor relative to the turbulent viscosity, used to correct its value, for low Reynolds numbers, given by Equation (23).

(23) α*=α∞*(α0*+RetRk1+RetRk).

In the Equation (23), Ret is the turbulent Reynolds number, which is calculated by Equation (24), and $R_{k}$ and $\alpha_{\infty }{}^{*}$ are model constants defined in Table 1. The parameters Ret e $\alpha_{0}{}^{*}$, can be obtained by Equations (24) and (25), as follows:

(24) Ret=ρkμω,

and

(25) $$\alpha_{0}{}^{*}=\frac{\beta_{i}}{3},$$

where,

(26) $$\beta_{i}=F_{1}\beta_{i,1}-\left(1-F_{1}\right)\beta_{i,2},$$

being ${\;\beta }_{i,1}$ and ${\;\beta }_{i,2}$ described in Table 1.

The term $F_{2}$, from Equation (22), is another coupling function between the models k-ω and k-ε, which is defined by Equation (27).

(27) $$F_{2}=\tan h\left(\theta_{2}{}^{2}\right),$$

where,

(28) $$\theta_{2}=\max\left[2\frac{\sqrt{k}}{0.09\omega d_{g}},\frac{500\mu }{\rho d_{g}{}^{2}\omega }\right].$$

The term S of Equation (22) is the magnitude of the fluid deformation rate, which is given by Equation (29).

(29) $$S\equiv \;\sqrt{2S_{\mathrm{ij}}S_{\mathrm{ij}}},$$

where,

(30) $$S_{\mathrm{ij}}=\frac{1}{2}\left(\frac{\partial u_{j}}{\partial X_{i}}+\frac{\partial u_{i}}{\partial X_{j}}\right).$$

The term $G_{k}$ of Equation (13) is defined by Equation (31) and represents the production of turbulent kinetic energy (k):

(31) $$G_{k}=-\rho \bar{{u^{\prime }}_{i}{u^{\prime }}_{j}}\frac{\partial u_{j}}{\partial X_{i}},$$

where the term $\bar{{u^{\prime }}_{i}{u^{\prime }}_{j}}$ represents Reynolds stresses, derived from turbulent flow.

The term $G_{\omega }$, from Equation (14), represents the production of the turbulent dissipation rate, and is given by:

(32) $$G_{\omega }=\;\alpha \frac{\omega }{k}G_{k},$$

where,

(33) α=α∞α*(α0+RetRω1+RetRω),

being $R_{\omega }$ a model constant (Table 1), and $\alpha_{\infty }$ is defined by Equation (34).

(34) $$\alpha_{\infty }=F_{1}\alpha_{\infty ,1}+\left(1-F_{1}\right)\alpha_{\infty ,2}.$$

In the Equation (34), $\alpha_{\infty ,1}$ and $\alpha_{\infty ,2}$ are given by:

(35) $$\alpha_{\infty ,1}=\frac{\beta_{i,1}}{\beta_{\infty }{}^{*}}-\;\frac{\lambda^{2}}{\sigma_{\omega ,1}\sqrt{\beta_{\infty }{}^{*}}},$$

and

(36) $$\alpha_{\infty ,2}=\frac{\beta_{i,2}}{\beta_{\infty }{}^{*}}-\;\frac{\lambda^{2}}{\sigma_{\omega ,2}\sqrt{\beta_{\infty }{}^{*}}},\;$$

where *λ* is a model constant (Table 3).

The term $Y_{k}$, from Equation (13) represents the dissipation of k, which is defined by Equation (37).

(37) $$Y_{k}={\rho \beta }^{*}k\omega ,$$

where,

(38) $$\beta^{*}=\beta_{i}{}^{*}\left[1+\xi^{*}\right].\;$$

The term $\beta_{i}{}^{*}$ of Equation (38) is given by:

(39) βi*=β∞*(415+(RetRβ)41+(RetRβ)4),

where $\xi^{*}$, $R_{\beta }$ and $\beta_{\infty }{}^{*}$ are constants in the model (Table 3).

The term $Y_{\omega }$ in Equation (14) represents the dissipation of ω, which can be calculated by Equation (40).

(40) $$Y_{\omega }={\rho \beta }^{*}\omega^{2}.$$

#### 2.3.2. Multiphase Model for Porous Media

In the Fluent software, the porous medium is modeled as a region containing elements of fluid, where the linear momentum equation is modified by the addition of a source term of dissipation. This source term is composed of two parts: One referring to the loss of viscous head (Darcy’s law, the first term on the right side of Equation (41)) and one referring to the loss of inertial head (the second term on the right of Equation (41)).

(41) $$S_{i}=-\left(\overset{3}{\underset{j=1}{\sum }}M_{\mathrm{ij}}{\mu v}_{j}+\overset{3}{\underset{j=1}{\sum }}N_{\mathrm{ij}}\frac{1}{2}\rho \left|v\right|v_{j}\right),$$

where $S_{i}$ is the source term for the i-th (x, y or z) momentum equation, |v| is the magnitude of the velocity, and M and N are prescribed matrices. This momentum sink contributes to the pressure gradient in the porous cell, creating a pressure drop that is proportional to the fluid velocity in the cell. Finally, homogeneous porous media were defined by Equation (42).

(42) $$S_{i}=-\left(\frac{\mu }{K_{i}\;}v_{i}+C_{2}\frac{1}{2}\rho \left|v\right|v_{i}\right),$$

where $K_{i}$ is the permeability, and $C_{2}$ is the inertial resistance factor, simplifying the matrices M and N as diagonal matrices with $\frac{1}{K_{i}\;}$ and $C_{2}$, respectively, occupying the values on the diagonals of the matrix. Given the low velocities developed in the volumes relative to the porous medium, the term referring to inertial resistance was neglected in this research.

#### 2.3.3. Boundary Conditions

(a) Module Input

The prescribed mass flow condition was established at the input of the separator module. Specifying the mass flow rate allows the total pressure to vary in response to the numerical solution. In this boundary condition, the absolute reference system, the direction of flow (normal to the inlet surface), the turbulence intensity, I = 5%, and the turbulent viscosity ratio $R_{\mu }$ = 10, given by Equations (43) and (44) were established:

(43) $$I\equiv \frac{u^{\prime }}{\bar{u}},$$

and

(44) $$R_{\mu }=\frac{\mu_{t}}{\mu },$$

where $u^{\prime }$ is the rate of velocity fluctuation, and $\bar{u}$ is the mean velocity of free flow. The values of k and ε are computed as a function of these parameters [30].

(b) Concentrate and Permeate Outlets

The prescribed pressure boundary condition was applied to the permeate and concentrate outlets. There was a zero gauge pressure at the outlets, that is, the ambient pressure. The pressure difference between the input of the separation module and the outputs (atmospheric pressure) drives the flow of produced water through the separation module.

(c) Module Wall and Membrane Surface

The wall conditions were used to connect the fluid and solid regions, surfaces of the module and membranes, in contact, externally, with the volume of the hull and, internally, with the domains related to permeate. Boundary conditions of no-slip and negligible roughness were applied to the external surfaces of the hull, surfaces of the supports, and in the ends of the membranes, hull, and permeate. For the internal and external surfaces of the membranes, the condition of interior wall (open surface) was used, which allowed the flow of produced water through the membranes, where, due to the source term relative to the porous medium, the phases were separated. Table 4 describes the limit regions of the module under study and their respective boundary conditions.

#### 2.3.4. Numerical Solution Method and Simulation Parameters

The discretization method applied in this work was that of finite volumes, the discretization method used by the software, which performs a balance of conservation equations for each control volume, which constitutes the numerical mesh.

For the pressure-velocity coupling, the Coupled algorithm, available in the ANSYS Fluent^®^ software, was used. The Coupled algorithm solves the continuity equations, based on the results of the calculation of the linear momentum conservation and the pressure, in a coupled way, which gives it, in relation to the segregated solution algorithms, a convergence range, with a smaller number of iterations.

The relaxation factors and the standard convergence criteria of ANSYS Fluent were adopted. The water and oil used in the numerical simulations had densities of 998.2 and 997 kg/m^3^, respectively, and dynamic viscosity of 0.001003 and 0.05 Pa·s, respectively. Based on the work of Cunha [26], the operating variables were standardized in mass flow rate ($\dot{m}$) 0.5 kg/s, oil concentration at the inlet, $(C_{0}$), 1 kg/m^3^, mean diameter of oil droplet ($d_{p}$), 63 μm, membrane permeability (K), 3 × 10^−13^ m^2^, and membrane porosity (ε), 30%.

### 2.4. Plans and Points Studied Inside the Module

**Figure 4:**
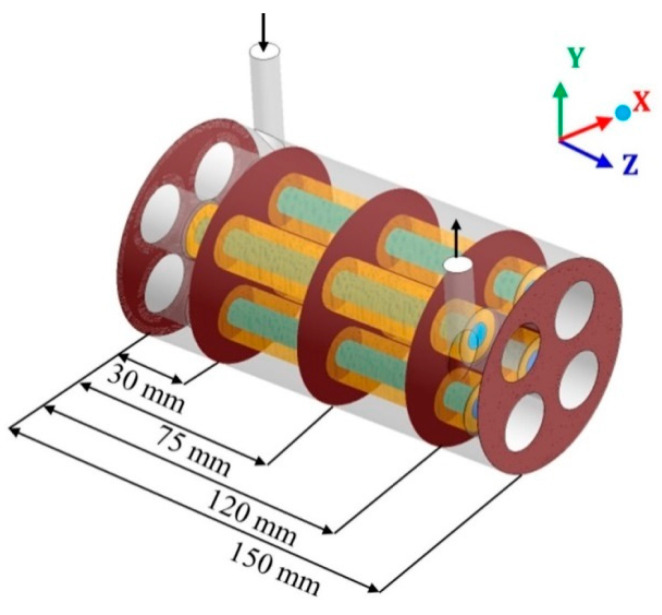
Transverse planes, in the hull region, chosen for data analysis.

**Figure 5:**
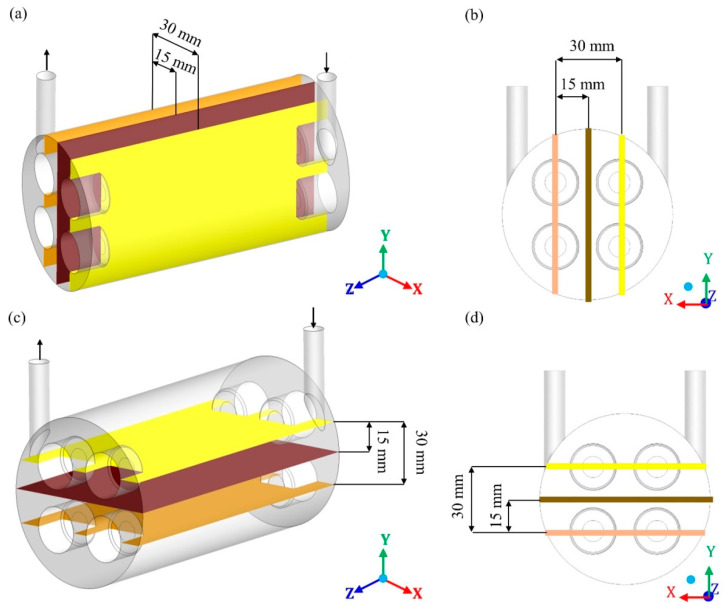
Vertical longitudinal planes (**a**,**b**) and horizontal longitudinal planes (**c**,**d**) in the hull region, chosen for data analysis.

**Figure 6:**
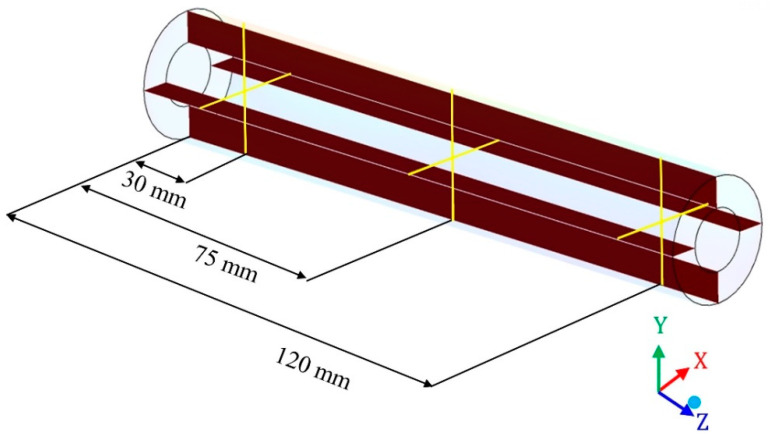
Vertical longitudinal planes and horizontal longitudinal planes in the membrane region, chosen for data analysis.

To study the characteristics of the filtration process during CFD simulation, in the module’s geometry was inserted different Cartesian planes, considering the x (transversal length), y (height), and z (longitudinal length) axis. Then, different planes were selected and evaluated at different points on the x, y, and z planes (Figure 4, Figure 5 and Figure 6).

The relative volumetric fraction of oil and the pressure were evaluated in the hull regions in the xy planes at z = 0, 30, 75, 120, and 150 mm; in the yz planes, at x = −15, 0, and 15 mm (where x = 0 mm corresponds to the center of the domain) and in the xz planes, at y = −15, 0, and 15 mm (where y = 0 mm corresponds to the center of the domain). The volumetric fraction of oil was also evaluated in the porous medium in the xy planes at z = 30, 75, and 120 mm, and throughout the permeate collecting tube. The mixing fluid velocity was evaluated throughout the porous medium and the permeate collecting tube.

The pressure was also evaluated inside the membranes, considering horizontal lines (x-axis): One line on the right (x ranging from −0.025 m to −0.020 m) and one on the left (x ranging from −0.005 m to −0.010 m), in such a way that the pressure was measured from the external surface (water / oil mixture side) to the inner membrane surface (permeate side); and vertical lines (y-axis): One line in the upper position (y ranging from 0.025 m to 0.020 m) and another in the lower position (y ranging from 0.005 m to 0.010 m). Both lines were evaluated at positions z = 30, 75 and 120 mm from the origin of the membrane (direction of the z axis).

## 3. Results and Discussions

### 3.1. Relative Volume Fraction of Oil in the Filtration Module

**Figure 7:**
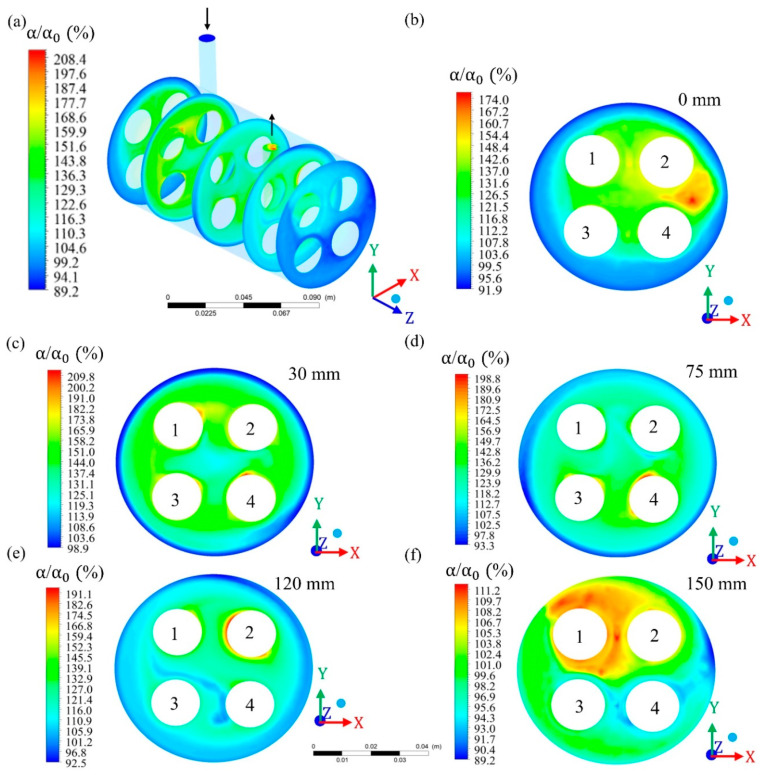
Field of relative oil volumetric fraction in different regions of the hull; (**a**) general view and in the xy plane at (**b**) z = 0 mm; (**c**) z = 30 mm; (**d**) z = 75 mm; (**e**) z = 120 mm; and (**f**) z = 150 mm, of the filtration module during the water/oil separation process.

Figure 7 illustrates the distribution of the volumetric fraction inside the module. In this analysis, the volumetric fraction of oil in the fluid is compared to the volumetric fraction of oil in the feed mixture. From the analysis of this figure, it can be seen that the oil volume fraction is minimal in the outermost region of the hull (α/$\alpha_{0}$ = 100%), indicating that the oil flows together with the feed mixture. Considering the hull region as a whole, it is observed that the oil volumetric fraction varied from 89.2% to 208.4%. Exactly at the origin of the hull (considering the xy plane when z = 0 mm), it is possible to observe a high volumetric fraction of oil (ranging from 154.4% to 174%) at the location of the module’s feed tube (Figure 7b), indicating that the oil is present in the water / oil mixture supplied to the system. Still in the plane of origin of the hull, it is possible to observe a lower oil concentration on the outer sides of the hull. To give you an idea, a volumetric fraction of 1% corresponds to an oil concentration of 9.97 kg/m^3^ or 9.97 g/L or 9970 ppm.

At 30 mm from the origin of the module (z = 30 mm), an oil volumetric fraction ranging from 98.9 to 209.8% is observed, more evenly distributed in relation to the plane at z = 0 mm and being more concentrated in the region between membranes and close to their surface, as shown in Figure 7c. From 30 mm, along the z axis, the maximum volume fraction of oil gradually reduced to the planes z = 75 mm (198.8%), z = 120 mm (191.1%,) and z = 150 mm (111.2%), indicating that the presence of oil in the hull is reduced, as the fluid goes towards the exit of the module (Figure 7d–f).

**Figure 8:**
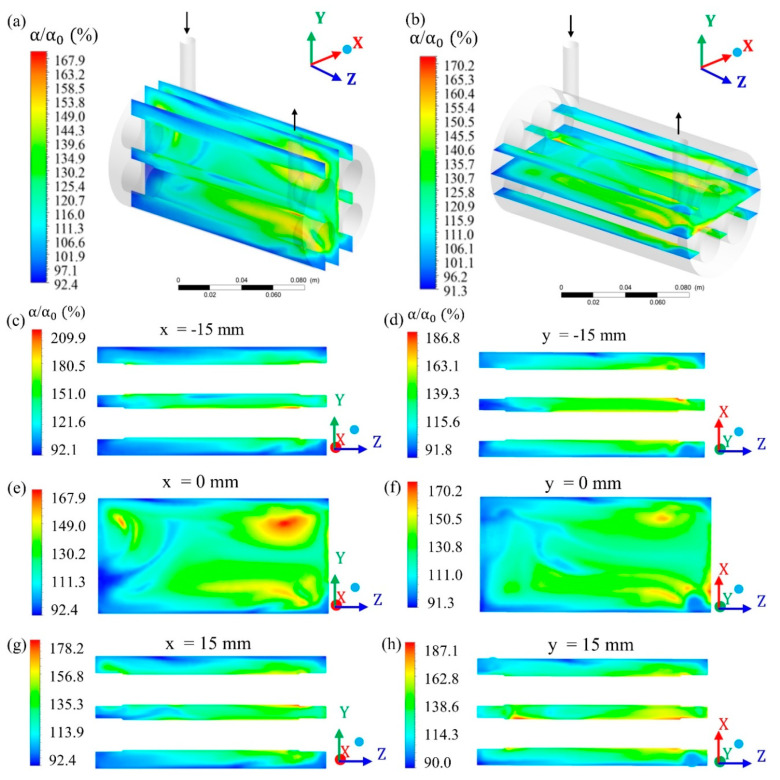
Field of relative oil volumetric fraction in different regions of the hull: (**a**) In the yz plane at x = −15 mm, x = 0 mm and x = 15 mm; (**b**) in the xz plane at y = −15 mm, y = 0 mm and y = 15 mm; (**c**) x = −15 mm; (**d**) y = −15 mm; (**e**) x = 0 mm; (**f**) y = 0 mm; (**g**) x = 15 mm and (**h**) y = 15 mm, of the filtration module during the water / oil separation process.

Figure 8 shows the distribution of the oil volume fraction at different planes. From the analysis of this figure, it can be seen that the average volumetric fractions of the oil in the parallel planes at different points along the x and y axes varied from 92.4% to 167.9% and from 91.3 to 170.2%, respectively (Figure 8a,b, respectively). Further, it can be seen that the lateral planes in the positions x = −15 mm and 15 mm (Figure 8c,g) and y = −15 mm and 15 mm (Figure 8d,h) present the largest maximum oil volume fractions when compared with their respective central planes, x = 0 mm and y = 0 mm (Figure 8e–f).

According to Habert et al. [32], Cunha [26], and Souza [27] this behavior occurs, because the oily fraction is retained mainly on the external surface of the porous medium, as the fluid flow enters the filtration module. Additional resistance to mass transfer occurs due to the establishment of a concentration gradient, leading to a decrease in the permeate flow. Therefore, this is a phenomenon that must be controlled and minimized, as it reduces the permeate flow and can affect the quality of the product [26].

**Figure 9:**
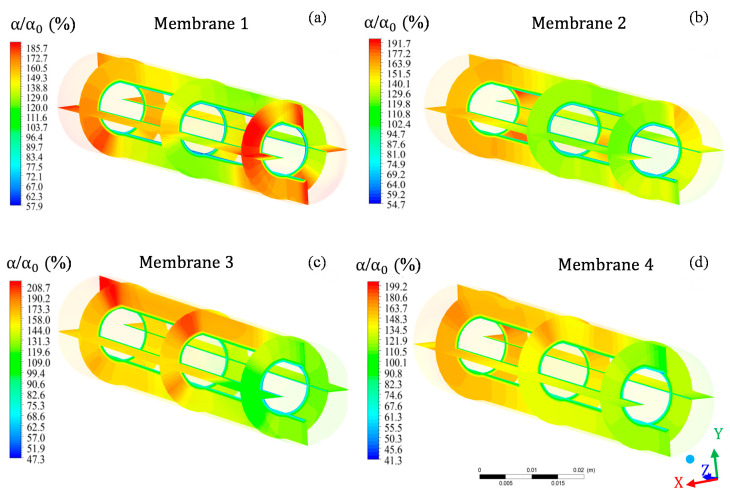
Field of relative oil volumetric fraction in different regions of membrane 1 (**a**), membrane 2 (**b**), membrane 3 (**c**), and membrane 4 (**d**) that make up the filtration module during the water/oil separation process.

When evaluating the relative oil volumetric fraction in the four porous membranes that make up the filtration module (Figure 9), a slight variation was observed in the maximum volumetric fractions of oil between the membranes as well as in the regions of greater oil presence in each membrane. For example, in membrane 1 (Figure 9a), the relative volumetric fraction of oil varied from 57.9% to 185.7%, while in membrane 3 (Figure 9c), the oil volumetric fraction varied from 47.3 to 208.7%. In addition, the region with the highest relative volumetric fraction of oil in membrane 1 was close to the exit region of the module, while, in membrane 3, this same region showed the lowest oil concentration. However, the relative oil volumetric fraction field was slightly similar between membranes 2 and 4 (Figure 9b,d), which, in turn, differed slightly from that observed in membranes 1 and 3. In general, the terminal region (close to the module exit) of membranes 2, 3, and 4 showed lower relative volume volumetric fractions of oil in relation to the region comprising 2/3 of the membrane measured from z = 0 mm.

**Figure 10:**
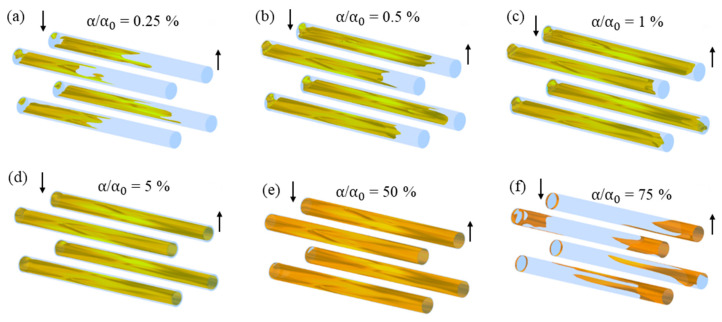
Iso-surfaces of the relative oil volumetric fraction in different regions of the permeated in the filtration module during the water/oil separation process: (**a**) $\alpha /\alpha_{0}=0.25\%$; (**b**) $\alpha /\alpha_{0}=0.5\%$; (**c**) $\alpha /\alpha_{0}=1\%$; (**d**) $\alpha /\alpha_{0}=5\%$; (**e**) $\alpha /\alpha_{0}=50\%$ and (**f**) $\alpha /\alpha_{0}=75\%$.

When analyzing the iso-surfaces of the relative oil volumetric fraction in the permeate (Figure 10), there is an absence of oil in the innermost region of the permeate (permeate outlet) or very low volumetric fraction of oil ($\alpha /\alpha_{0}$ = 0.25%), located mainly in the part of the permeate collecting tube, which is close to the feeding region (Figure 10a). On the other hand, in the permeate near the membrane interface, a higher oil concentration is observed ($\alpha /\alpha_{0}$ = 75%), located mainly in the permeate outlet (Figure 10f). In this sense, the fraction of oil retained gradually increased to more distant regions of the permeate collecting tube.

In general, the accumulation of relative oil fractions in the hull, membranes and permeate occurred due to the drag force and the tangential flow direction, which carries the retained particles to the terminal end of the module.

### 3.2. Pressure in the Filtration Module

**Figure 11:**
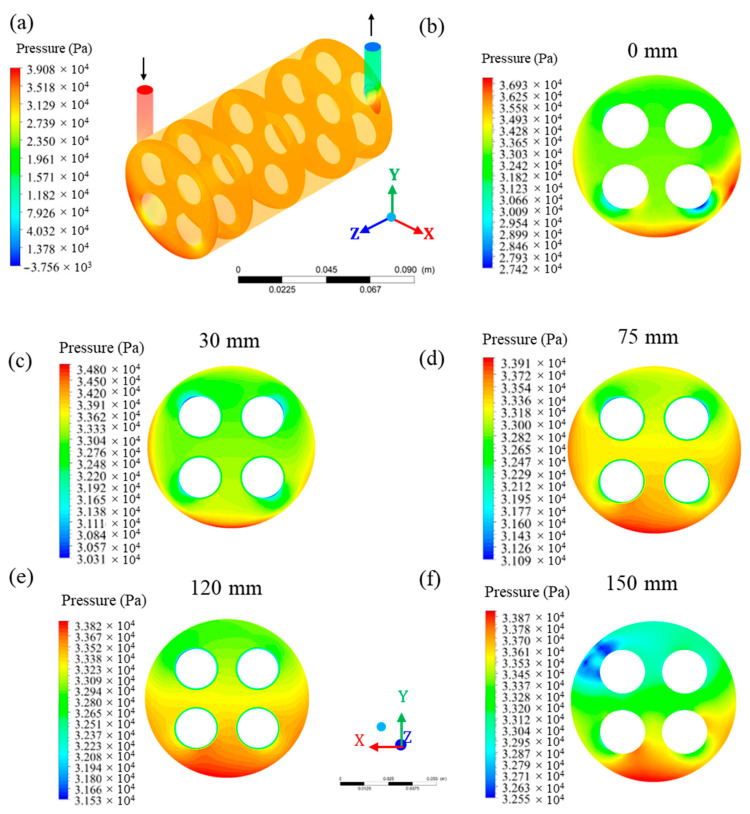
Pressure field in different regions of the hull. (**a**) Overview and in the xy plane at z = 0 mm (**b**), z = 30 mm (**c**), z = 75 mm (**d**), z = 120 mm (**e**), and z = 150 mm (**f**), of the filtration module during the water / oil separation process.

Figure 11 shows the pressure distribution in the hull region. From the analysis of this figure, it can be seen that the pressure in the hull was higher in the region of entry and close to this region, reaching a maximum average pressure of 39.08 kPa. On the other hand, at the permeate outlet, the lowest pressure values were observed. This information is ratified when comparing the maximum pressure in the xy plane at z = 0 mm (inlet; Figure 11b) with z = 150 mm (inlet; Figure 11f). Comparing the results obtained in the different planes along the x-axis, at positions z = 0, 30, 75, 120, and 150 mm, a reduction in system pressure is observed that occurs gradually from the entrance to the module outlet.

Observing the transverse planes at positions z = 0, 30, 75, 120, and 150 mm of the hull, it is noted that the pressure tends to be greater in the lower region of the hull, especially in the region below and between membranes 3 and 4. In the transverse planes in the positions z = 0 mm and z = 30 mm, higher pressures (36.93 and 34.80 kPa, respectively) are observed, located in the lower and lateral regions, while the lowest pressures occur close to the membranes (27.42 and 30.31 kPa, respectively). In the transverse planes in the positions z = 75 mm and z = 120 mm, the minimum and maximum pressure varied from 31.09 to 33.91 kPa and from 31.53 to 33.82 kPa, respectively. In both planes, higher and more distributed pressures can be observed in the lower region of the hull, especially in the region close to the surface of the membranes. In the transverse plane in the position z = 150 mm, the pressure varied from 32.55 to 33.87 kPa, with the highest pressure being located in the lower region, and the lowest pressure, located in the permeate exit region.

**Figure 12:**
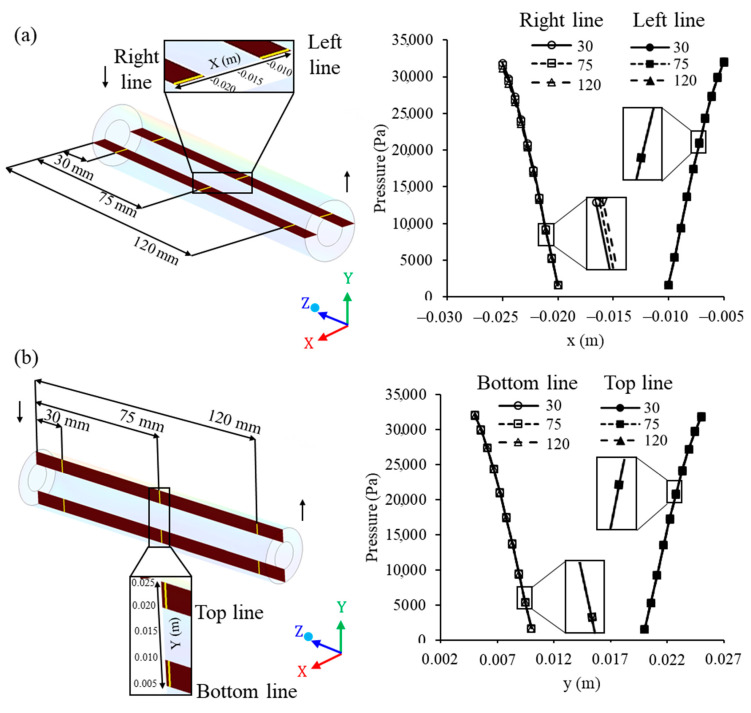
Pressure on the membrane at different (**a**) horizontal and (**b**) vertical points (z = 30, 75, and 120 mm) of the filtration module during the water/oil separation process.

The pressure along the membrane was assessed in two horizontal lines (x-axis): One line on the right (x ranging from −0.025 to −0.020 m) and another line on the left (x ranging from −0.005 to −0.010 m), in such a way that this parameter was measured from the outer surface (water/oil mixture side) to the inner membrane surface (permeate side) and evaluated at z = 30, 75, and 120 mm, from the origin of the membrane (direction of the z axis), as shown in Figure 12a. Further, transmembrane pressure was also assessed on two vertical lines (y-axis): A line in the upper position (y ranging from 0.020 to 0.025 m) and another in the lower position (y ranging from 0.005 to 0.010 m), also at z = 30, 75, and 120 mm, from the origin of the membrane (direction of the z axis), as illustrated in Figure 12b.

The pressure in the membrane was practically constant along the z axis (longitudinal length) of the membrane, observed by the similar values in the positions z = 30, 75 and 120 mm of the entrance, and in all the right and left lines (direction x) and top and bottom (y direction). This behavior indicates an efficient system to maintain the same pressure throughout the filtration membrane module. In turn, the pressure differential in the system is a determining factor, to ensure the flow through the membrane, keeping the high filtration process efficiency for a longer period of time [8,11].

### 3.3. Mixing Speed in the Filtration Module

**Figure 13:**
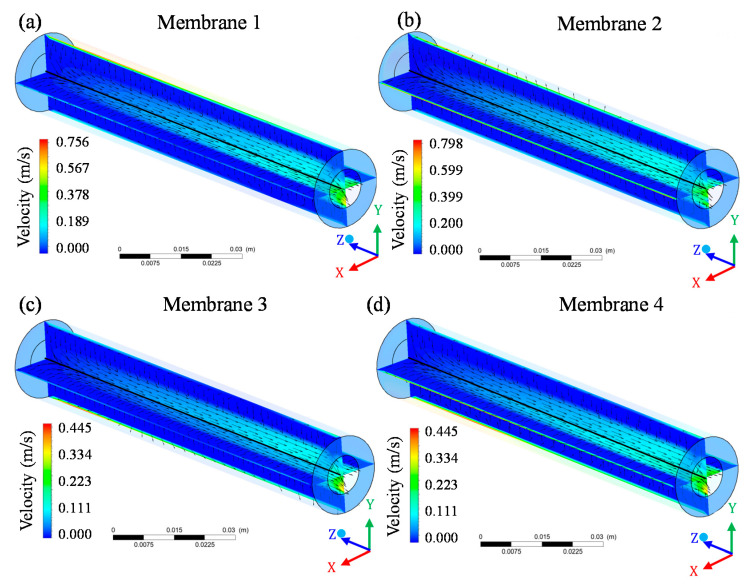
Velocity distribution of the fluid mixture inside the membranes and permeated in the filtration module during the water/oil separation process: (**a**) membrane 1; (**b**) membrane 2; (**c**) membrane 3 and (**d**) membrane 4.

Figure 13 illustrates the velocity distribution of the mixture at different planes within the membrane. Analyzing this figure, it can be seen that the velocity at the surface of the membranes increased in the direction of entry–exit, that is, the farther from the region of entry of the module, the greater the fluid mixture velocity on the membrane surface. In fact, the velocity at the initial position of the membrane surface was zero or very close to zero. This same behavior was observed in the 4 membranes inside the module, however the maximum velocity in membranes 1 and 2 (Figure 13a,b) was higher than the maximum velocity in membranes 3 and 4 (Figure 13c,d).

The increase in velocity along the membrane surface occurs because the tangential flow runs through the free space inside the module parallel to the membranes surfaces and in the form of a vortex. Thus, at various points along the z-axis, the behavior of the fluids present in the module causes an increase in the fluid mixture velocity that runs parallel to the membrane surfaces. These results are in line with those reported by Cipollina et al. [33], when simulating the flow and temperature fields in a region of a membrane module in spirals, with double layer filament spacer, using CFD tools and considering a 25 × 25 × 3 mm size domain in x, y and z directions, respectively.

**Figure 14:**
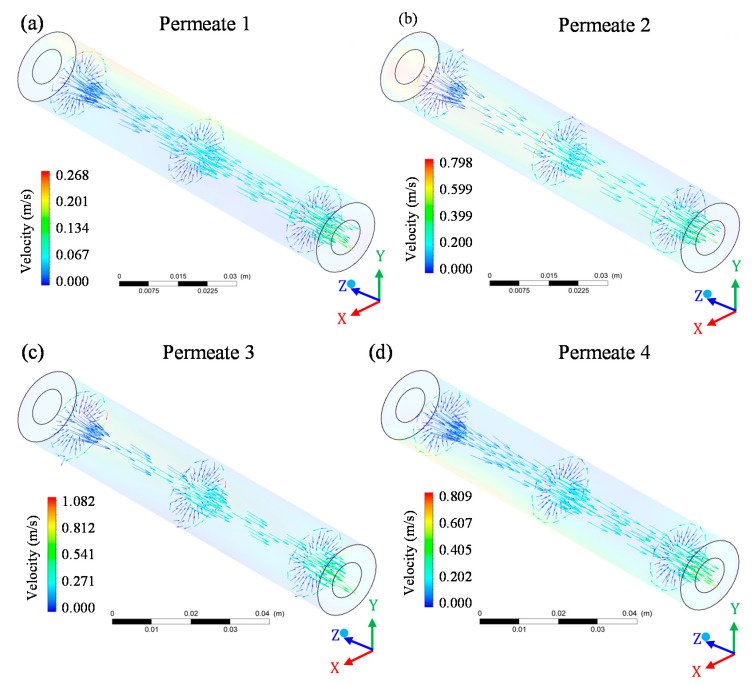
Vector velocity field of permeate in the four membranes of the filtration module during the water/oil separation process: (**a**) permeate 1; (**b**) permeate 2; (**c**) permeate 3 and (**d**) permeate 4.

The permeate velocity inside the membranes (permeate collecting tube) gradually increased from the entrance region to the exit area of the system (Figure 14). This behavior was common throughout the flow and occurs because, along the longitudinal axis of the membrane (z-axis), the flow is continuously filtered, crossing the membrane and feeding the permeate tube, thereby increasing the mass flow rate of the permeate. Thus, the more mass flow rate of the permeate is added to the collection tube, an increase in the total flow velocity is expected.

Although membrane 1 showed a high maximum velocity on the surface, especially when compared to membranes 3 and 4, the maximum permeate velocity for membrane 1 (Figure 14a) was at least 4 times lower than that observed for membranes 3 (Figure 14c) and 4 (Figure 14d). The decrease in the fluid flow through the membranes during the filtration process occurred due to the obstruction of the membrane pores [26,27,32,34] as well as the formation of a concentration polarization layer, which is established on the surface of membranes and which can reduce the permeate volume per unit time [15].

**Figure 15:**
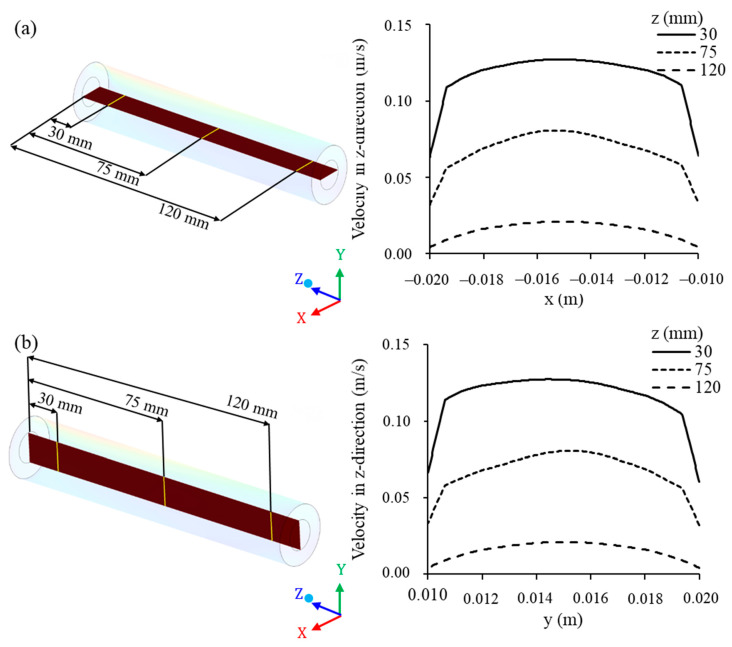
Axial permeate velocity at different (**a**) horizontal and (**b**) vertical points along the membrane collecting tube, at z = 30, 75, and 120 mm, of the filtration module during the water/oil separation process.

The fluid mixture velocity in the membrane collecting tube (Figure 15) was evaluated in horizontal lines (x-axis, ranging from −0.020 m to −0.010 m) and vertical lines (y-axis, ranging from 0.010 m to 0.020 m) at the positions z = 30, 75, and 120 mm, from the origin of the module (direction of the z axis), as shown in Figure 15a,b. From the analysis of this figure, it appears that the fluid velocity was low at the position z = 30 mm, from the origin of the membrane, increasing in the direction of the z axis, as observed for the positions of z = 75 mm and z = 120 mm. These changes in the fluid mixture velocity inside the tube were very similar between the horizontal and vertical lines.

In the center of the permeate collecting tube (centroid of the x and y axes, −0.015 and 0.015 m, respectively), the permeate velocity was higher and decreased as it approached the inner surface of the membrane (region of membrane–permeate contact). It can be seen that the velocity profile is developing, from the behavior of a laminar flow (parabolic velocity profile) to a turbulent flow (flatter velocity profile).

## 4. Conclusions

Currently, ceramic membrane has been considered one of the most promising technologies in the treatment of contaminated water. This is due to its excellent mechanical, chemical, and biological characteristics. In this research, simulations related to the process of separating a mixture of water and oil using a ceramic membrane module and the CFD technique were carried out under different operating conditions. From the results obtained, it can be concluded that:

(a) The proposed mathematical modeling proved be effective in predicting the fluid dynamic behavior of the oil and water phases inside the separation module in the separation process;
(b) the new configuration of the ceramic membrane module showed major performance compared to one ceramic membrane alone operating in the same conditions;
(c) the volumetric fraction of oil increases from the entrance of the module to the exit of the concentrate and decreases inside the membrane, from the external surface to the exit of the permeate;
(d) the pressure inside the module decreases from the feed inlet to the outlets of the concentrate and permeate, being higher in the vicinity of the lower inner surface of the hull; and
(e) the transmembrane pressure was almost constant along the longitudinal length of the membranes, indicating an efficient system in maintaining the same pressure throughout the filtration module, favoring the mass transfer and oil retention.

Under the practical point of view, the contribution of this research is clearly seen: The proposed membrane module can be used in the analysis of different materials (ceramic or polymeric membranes, oil and water or solid particle and water, for examples); at different operating conditions of feed flow rate (low or high), fluid mixture composition (high or low solute concentration), membrane physical parameters (porosity and permeability), membrane geometrical parameters (length, diameter, and wall thickness), and at different liquid–liquid and liquid–solid separation processes (microfiltration, ultrafiltration, nanofiltration, and reverse osmosis). This way, it is possible to analyze different physical situations and to assist industrial engineers in making appropriate and safety decisions on this topic, according to the level of filter required and type of applications.

## Figures and Tables

**Table 1:** Geometric dimensions of the shell-tube separation module.

Parameters	Module Dimensions (mm)
Separator module length	150
Useful length of each membrane	114
Length of each membrane support	18
Diameter of separation module	80
Inner diameter of each membrane	10
Outer diameter of each membrane	20
Outside diameter of each support	22
Inlet duct diameter	10
Outlet duct diameter	10
Inlet duct length	60
Outlet duct length	60
Membrane thickness	10

**Table 2:** Quality parameters of the constructed meshes.

Mesh	Orthogonality (ANSYS)		Deformation (ANSYS)		Number of Elements
	Minimum	Average	Maximum	Average	
1	0.205	0.897	0.848	0.203	218,704
2	0.146	0.897	0.872	0.196	508,325
3	0.192	0.904	0.847	0.179	853,536

**Table 3:** Turbulence model constants k-ω SST (shear-stress transport).

Constants	Numeric Value
$\sigma_{k,1}$	1.176
$\sigma_{k,2}$	1.0
$\sigma_{\omega ,1}$	2.0
$\sigma_{\omega ,2}$	1.168
$R_{k}$	6
$\alpha_{\infty }{}^{*}$	1
${\;\beta }_{i,1}$	0.075
$\beta_{i,2}$	0.0828
$R_{\omega }$	2.95
λ	0.41
$\xi^{*}$	1.5
$R_{\beta }$	8
$\beta_{\infty }{}^{*}$	0.09

* Source: ANSYS Fluent Theory Guide [30].

**Table 4:** Limit regions of the modules and boundary conditions.

Regions	Boundary Condition
Separator inlet	Prescribed mass flow
Separator outlets (permeate 1, 2, 3, and 4 and concentrate)	Prescribed pressure
Internal and external surfaces of membranes	Interior (*Software*)
External surfaces of the hull	Wall (zero velocity)
Support surfaces	Wall (zero velocity)
Membrane ends, hull and permeate	Wall (zero velocity)

## References

[1] Jiménez S., Micó M.M., Arnaldos M., Medina F., Contreras S. (2018). State of the art of produced water treatment. Chemosphere.

[2] Huang K.Z., Xie Y.F., Tang H.L. (2019). Formation of disinfection by-products under influence of shale gas produced water. Sci. Total Environ..

[3] Fakhru’l-Razi A., Pendashteh A., Abdullah L.C., Biak D.R.A., Madaeni S.S., Abidin Z.Z. (2009). Review of technologies for oil and gas produced water treatment. J. Hazard. Mater..

[4] Igunnu E.T., Chen G.Z. (2014). Produced water treatment technologies. Int. J. Low-Carbon Technol..

[5] Khan F.A., Hisham S., Ghasemi M. (2019). Oil field produced water recovery and boosting the quality for using in membrane less fuel cell. SN Appl. Sci..

[6] (2011). CONAMA Resolution No 430 Art. 4. http://www.adasa.df.gov.br/images/stories/anexos/8Legislacao/Res_CONAMA/Resolucao_CONAMA_430_2011.pdf.

[7] Crini G., Lichtfouse E. (2019). Advantages and disadvantages of techniques used for wastewater treatment. Environ. Chem. Lett..

[8] Yu L., Han M., He F. (2017). A review of treating oily wastewater. Arab. J. Chem..

[9] Jepsen K., Hansen L., Mai C., Yang Z. (2016). Challenges of membrane filtration for produced water treatment in offshore oil & gas production. Proceedings of the OCEANS 2016 MTS/IEEE Monterey.

[10] Syarifah Nazirah W.I., Norhaniza Y., Farhana A., Nurasyikin M. (2017). A review of oilfield wastewater treatment using membrane filtration over conventional technology. Malays. J. Anal. Sci.

[11] Padaki M., Murali R.S., Abdullah M.S., Misdan N., Moslehyani A., Kassim M.A., Hilal N., Ismail A.F. (2015). Membrane technology enhancement in oil–water separation. A review. Desalination.

[12] Almojjly A., Johnson D., Hilal N. (2019). Investigations of the effect of pore size of ceramic membranes on the pilot-scale removal of oil from oil-water emulsion. J. Water Process Eng..

[13] Kwasny J.A., Krylow M., Balcerzak W. (2018). Oily wastewater treatment using a zirconia ceramic membrane-a literature review. Arch. Environ. Prot..

[14] da Biron D.S., Zeni M., Bergmann C.P., dos Santos V. (2017). Analysis of Composite Membranes in the Separation of Emulsions Sunflower oil/water. Mater. Res..

[15] Cheng R.Z., Qiu L.P., Liu G.C., Qiu Q., Han Q., Wang Y. (2018). Application Prospect of Ceramic Membrane Coupling Process in Refinery Wastewater. MS E.

[16] Fard A.K., Bukenhoudt A., Jacobs M., McKay G., Atieh M.A. (2018). Novel hybrid ceramic/carbon membrane for oil removal. J. Membr. Sci..

[17] Mohammadi T., Kazemimoghadam M., Saadabadi M. (2003). Modeling of membrane fouling and flux decline in reverse osmosis during separation of oil in water emulsions. Desalination.

[18] Cui J., Zhang X., Liu H., Liu S., Yeung K.L. (2008). Preparation and application of zeolite/ceramic microfiltration membranes for treatment of oil contaminated water. J. Membr. Sci..

[19] Li M., Zhao Y., Zhou S., Xing W., Wong F.-S. (2007). Resistance analysis for ceramic membrane microfiltration of raw soy sauce. J. Membr. Sci..

[20] Zhou J., Chang Q., Wang Y., Wang J., Meng G. (2010). Separation of stable oil–water emulsion by the hydrophilic nano-sized ZrO_2_ modified Al_2_O_3_ microfiltration membrane. Sep. Purif. Technol..

[21] Hua F.L., Tsang Y.F., Wang Y.J., Chan S.Y., Chua H., Sin S.N. (2007). Performance study of ceramic microfiltration membrane for oily wastewater treatment. Chem. Eng. J..

[22] Hubadillah S.K., Tai Z.S., Othman M.H.D., Harun Z., Jamalludin M.R., Rahman M.A., Jaafar J., Ismail A.F. (2019). Hydrophobic ceramic membrane for membrane distillation: A mini review on preparation, characterization, and applications. Sep. Purif. Technol..

[23] Shirazian S., Rezakazemi M., Marjani A., Moradi S. (2012). Hydrodynamics and mass transfer simulation of wastewater treatment in membrane reactors. Desalination.

[24] Shirazian S., Pishnamazi M., Rezakazemi M., Nouri A., Jafari M., Noroozi S., Marjani A. (2012). Implementation of the finite element method for simulation of mass transfer in membrane contactors. Chem. Eng. Technol..

[25] Rezakazemi M., Shirazian S., Ashrafizadeh S.N. (2012). Simulation of ammonia removal from industrial wastewater streams by means of a hollow-fiber membrane contactor. Desalination.

[26] Cunha A.L. (2014). Treatment of Effluents from the Petroleum Industry via Ceramic Membranes—Modeling and Simulation. Doctoral Thesis.

[27] Souza J.S. (2014). Theoretical Study of the Microfiltration Process in Ceramic Membranes. Doctoral Thesis.

[28] Magalhaes H.L.F., de Lima A.G.B., de Farias Neto S.R., Alves H.G., de Souza J.S. (2017). Produced water treatment by ceramic membrane: A numerical investigation by computational fluid dynamics. Adv. Mech. Eng..

[29] Rezakazemi M. (2018). CFD simulation of seawater purification using direct contact membrane desalination (DCMD) system. Desalination.

[30] ANSYS, Inc. (2013). ANSYS Fluent Theory Guide 15.0.

[31] Kleinstreuer C. (2003). Two-Phase Flow: Theory and Applications.

[32] Habert A.C., Borges C.P., Nobrega R. (2006). Escola Piloto em Engenharia Química: Processos de separação com membranas. Escola Piloto em Engenharia Química.

[33] Cipollina A., Di Miceli A., Koschikowski J., Micale G., Rizzuti L. (2009). CFD simulation of a membrane distillation module channel. Desalin. Water Treat..

[34] Rai P., Rai C., Majumdar G.C., DasGupta S., De S. (2006). Resistance in series model for ultrafiltration of mosambi (*Citrus sinensis* (L.) Osbeck) juice in a stirred continuous mode. J. Membr. Sci..

